# Genetic Inactivation of European Sea Bass (*Dicentrarchus labrax* L.) Eggs Using UV-Irradiation: Observations and Perspectives

**DOI:** 10.1371/journal.pone.0109572

**Published:** 2014-10-20

**Authors:** Julie Colléter, David J. Penman, Stéphane Lallement, Christian Fauvel, Tanja Hanebrekke, Renate D. Osvik, Hans C. Eilertsen, Helena D’Cotta, Béatrice Chatain, Stefano Peruzzi

**Affiliations:** 1 Cirad, Persyst, UMR Intrepid, Campus International de Baillarguet, Montpellier, France; 2 Ifremer, Laboratoire de Recherche Piscicole en Méditerranée, Station Expérimentale d’Aquaculture, Palavas Les Flots, France; 3 University of Stirling, Institute of Aquaculture, Stirling, Scotland; 4 Department of Arctic and Marine Biology, Faculty of Biosciences, Fisheries and Economics, University of Tromsø, Tromsø, Norway; Temasek Life Sciences Laboratory, Singapore

## Abstract

Androgenesis is a form of uniparental reproduction leading to progenies inheriting only the paternal set of chromosomes. It has been achieved with variable success in a number of freshwater species and can be attained by artificial fertilization of genetically inactivated eggs following exposure to gamma (γ), X-ray or UV irradiation (haploid androgenesis) and by restoration of diploidy by suppression of mitosis using a pressure or thermal shock. The conditions for the genetic inactivation of the maternal genome in the European sea bass (*Dicentrarchus labrax* L.) were explored using different combinations of UV irradiation levels and durations. UV treatments significantly affected embryo survival and generated a wide range of developmental abnormalities. Despite the wide range of UV doses tested (from 7.2 to 720 mJ.cm^−2^), only one dose (60 mJ.cm^−2^.min^−1^ with 1 min irradiation) resulted in a small percentage (14%) of haploid larvae at hatching in the initial trials as verified by flow cytometry. Microsatellite marker analyses of three further batches of larvae produced by using this UV treatment showed a majority of larvae with variable levels of paternal and maternal contributions and only one larva displaying pure paternal inheritance. The results are discussed also in the context of an assessment of the UV-absorbance characteristics of egg extracts in this species that revealed the presence of gadusol, a compound structurally related to mycosporine-like amino acids (MAAs) with known UV-screening properties.

## Introduction

Androgenesis is a form of uniparental development in which the nuclear genetic material is entirely of paternal origin. Androgenesis can be artificially induced in fish using a variety of methods, mostly involving the inactivation of the egg genome by UV or ionizing irradiation (see reviews [1]–[4]). Most commonly, genetically inactivated eggs are fertilized with conspecific haploid sperm and the paternal genome is doubled by suppression of the first cleavage using thermal or pressure shocks, leading to the production of doubled haploids (DH). Alternative techniques involve the use of diploid sperm from tetraploid fish [5], [6] or dispermic egg activation via fusion of sperm nuclei [7], which make the diploidization step unnecessary. Despite their lower penetrance [8], UV-rays have been most widely employed as being more manageable, less damaging to the eggs and far less prone to produce chromosome fragments than ionizing irradiations [9]–[12]. DH androgenetics are expected to be homozygous at all loci as they are produced by duplication of a single set of paternal chromosomes. This condition adversely affects the development and viability of DH embryos because of the expression of homozygous deleterious alleles. The viability of DH androgens can be further reduced because of potential damage caused by irradiation and physical shock to egg mitochondrial DNA (mtDNA) and other cytoplasm constituents [4], [13].

Androgenesis is a useful tool for e.g. the study of sex determination [3], [14], [15], the production of homozygous clones for research purposes [16]–[19], the preservation and recovery of unique strains or endangered species from cryopreserved sperm [20] and the study of physiological effects of mitochondrial variations [21]. Androgenesis has been achieved with variable success in a number of freshwater species including Nile tilapia *Oreochromis niloticus* [22], [23], zebrafish *Danio rerio* [24], [25], common carp *Cyprinus carpio* [26], [27] and rainbow trout *Oncorhynchus mykiss* [28], [29], as reviewed by Komen and Thorgaard [4].

The complete inactivation of the maternal genome is the prerequisite for the successful induction of androgenesis [24], [30]–[33]. Different techniques have been used to verify the androgenetic status of experimental fish such as embryo and larval morphology, nuclear DNA content, chromosomes counts, microsatellite markers or fingerprinting [23], [27], [33]–[36].

The European sea bass is a species of considerable economic importance in the Mediterranean and North East Atlantic regions both from the aquaculture and fishery perspectives. Several studies aimed at improving its culture performance have highlighted the need for better characterization of this species [37], [38]. A range of isogenic clonal lines would allow comparison over time and under different ambient conditions, estimation of genetic correlations, detection of genotype-by-environment interactions and estimation of phenotypic plasticity for complex traits [4]. Such lines would also be advantageous for other studies such as gene mapping, genome sequencing, epigenetic effects and detection of important quantitative trait loci (QTLs) for culture and research purposes.

Clonal lines of sea bass could be more rapidly achieved by androgenesis since some precocious males mature earlier as one year of age [39]. Meiotic and mitotic gynogenesis have been successfully induced in the European sea bass [40], [41] but no attempt at androgenesis has been reported for this species to date and to our knowledge androgenesis has not been reported in any marine species.

In this work, we explore the conditions for the genetic inactivation of the maternal genome in the European sea bass using UV-irradiation, with the future goal of producing viable diploid androgenetics. Given that the eggs of several marine teleosts [42], [43], including some Mediterranean species [44], contain variable levels of mycosporine-like amino acids (MAAs, notably gadusol) that provide protection against ambient UV-irradiation, particularly in small, transparent and positively buoyant fish eggs [45], a preliminary assessment of the UV-absorbance characteristics of egg extracts in this species is also described.

## Materials and Methods

### Ethics

This study was carried out in strict accordance and agreement with the recommendations of the Animal Care Committee of France. All experiments were performed under the official animal experimentation license of B. Chatain (C 34-41, Level 1) approved by the Ministry for Agriculture, Agroalimentation and Forestry and in a certified laboratory (C 34-192-6) approved by the same Ministry. All experimenters hold an animal experimentation license level 2. All biometries were performed under phenoxyethanol anesthesia (200 ppm) in order to reduce stress during manipulations of fishes. No surgery or suffering manipulations were performed on fishes.

### Experimental design

In order to investigate the efficiency of UV rays at inactivating the maternal genome and induce haploid androgenesis in sea bass we exposed pools of eggs (mixed from different females) to different incident UV-doses and durations followed by activation with normal sperm. To optimize androgenesis treatment using UV rays, irradiation was provided by two sources, from above and below the eggs [34], and mechanical stirring was also used to assure homogenous egg irradiation [27]. In order to prevent DNA photoreactivation, egg irradiation and early incubation were completed under total darkness. Observations on embryo larval morphology and survival were used as indicators of treatment conditions and supported by nuclear DNA content estimations of surviving larvae in each experiment. For the confirmation of parental inheritance in putative androgenetic larvae, different batches of eggs were exposed, in a separate experiment, to the best performing UV-conditions from the initial experiments, fertilized with untreated sperm and the resulting larvae genotyped using a set of microsatellite markers (see section 2.6). Newly hatched larvae in the European sea bass are very small, so cytometric analyses and genotyping could not be performed on the same individuals. As a positive control, the efficiency of the purpose built UV device was verified by using the eggs of a model species, the Nile tilapia, and following published procedures for the induction of haploid androgenesis in this species (File S1). Finally, egg extracts were analyzed through spectrophotometry and High Performance Liquid Chromatography (HPLC) to look for possible UV-screening compounds (sections 2.8 and 2.9).

### Broodstock and gamete collection

The sea bass broodstock (around 120 females and 40 males) was composed of domesticated and selected fish of West-Mediterranean and Atlantic origin held at the Ifremer Experimental Aquaculture Station (Palavas-les-Flots, France). Fish were aged 4 to 6 years and weighted 1 to 5 kg, they were kept in recirculated systems (8 m^3^ tanks, rate of O_2_ enriched water renewal: 250 L.h^−1^, constant small air flow) maintained under natural conditions of temperature and photoperiod (43° 31′ 40 N, 3° 55′ 37 E) and fed commercial diets (NeoRepro, Le Gouessant, France). Running males were recognized by gentle abdominal pressure and held in an easy handling tank. Female maturation stage was assessed in ovarian biopsies obtained by introducing a thin catheter (Pipelle de Cornier, Laboratoire CCD, Paris, France) in the genital orifice. Oocyte diameter and germinal vesicle migration were analyzed after addition of a clearing agent (glacial acetic acid, formaldehyde, ethanol in a ratio 1∶3∶6) using a profile projector (Nikon V12). Females at the correct stage of development [46] received a single dose (10 µg.kg^−1^) of Luteinizing Hormone Releasing Hormone analogue (LHRHa, Sigma, France) in order to induce final maturation and ovulation. The treated fishes were isolated in individual thermoregulated (13°C) tanks (1.5 m^3^, 17 L.h^−1^ water renewal, low air flow) and 72 h after female hormonal stimulation, ovulated oocytes were collected by abdominal pressure. Sperm was drawn from the genital papilla under abdominal pressure, using 5 ml syringes, after carefully wiping off water from the genital papilla and avoiding contamination with urine and/or faeces, and held at 4°C until use. At this stage, caudal fin clips were taken from parent fish and stored in absolute ethanol for future genetic analyses. Equal volumes of suitable eggs from 3–5 females were pooled in a single 1 L beaker for further treatment in each experiment.

### UV-irradiation of eggs

The UV irradiation device was composed of eight UV germicidal lamps (12 W, 254 nm, Vilber-Lourmat, Marne-la-Vallée, France) fixed above and below (four lamps each) a quartz plate mechanically stirred throughout irradiation. Small aliquots of eggs (3 ml, around 3000 eggs) were poured into 8.5 cm diameter quartz Petri dishes containing 3 ml of artificial extender SGSS (Seabass Gamete Short term Storage) made of Storefish (IMV Technologies, France) complemented with pyruvate and glutamine at 0.6 and 3 mg.ml^−1^ respectively (C. Fauvel, pers. comm.), to form a single layer of eggs: the quartz plate and Petri dishes (SARL NH Verre, Puechabon, France) were employed to maximize UV transmission during treatments. Incident UV dose rates were calculated as the addition of the measured doses from above and below. The eggs were irradiated using different incident UV dose rates (7.2, 13.2, 28.8, 42, 54, 60 or 72 mJ.cm^−2^.min^−1^) and durations (0.5–12 min) according to the following combinations: low dose rates (7.2–28.8 mJ.cm^−2^.min^−1^) for 1, 2, 4, 6, 8, 10 and 12 min, high dose rates (42–72 mJ.cm^−2^.min^−1^) for 0.5, 1, 2, 4, 6, 8 and 10 min and additional 0.75 and 1.25 min treatments for 60 mJ.cm^−2^.min^−1^. Cumulative irradiation doses were calculated by multiplying incident dose rate by duration of irradiation. The lamps were switched on at least 30 min before the onset of irradiation and UV incident dose was verified at the beginning and at the end of each experiment using a VLX-3W radiometer (Vilber-Lourmat), checking both upper and lower UV sources. Egg fertilization was performed just after irradiation by adding 80 µl sperm diluted (1∶4) in SGSS and 3 ml sea water (14°C, 35‰). Each experiment was replicated three times using the same oocyte pool and each UV dose was tested twice using different pools of oocytes. Control groups consisted of fertilized eggs that were not irradiated. They were handled and fertilized as above (apart from UV irradiation). All experiments were performed under total darkness in a temperature controlled room maintained at 14°C. Shortly after fertilization, control and treated eggs were incubated separately in individual 2 L tanks in a dedicated recirculated water system (temperature: 14–14.5°C; salinity: 35–36‰) until hatching. All tanks were maintained in darkness for the first 24 h of incubation before being exposed to natural light conditions.

### Estimation of embryonic and larval survival

To characterize embryo development and estimate survival, three different countings were made using sub-samples of approximately 200 eggs collected from each incubator. The first counting, realized 2–4 hours post fertilization (hpf) was used to assess fertilization rate at 4–8 cells stage. The second and third countings were performed at 50 and 74 hpf, respectively, to assess further embryonic development. All observations were made using a dissecting microscope (M3C, Wild Heerbrugg, Switzerland) and representative photomicrographs were taken using a Stemi 2000-C stereomicroscope (Carl Zeiss, Germany) equipped with a ProgResC5 camera device (Jenoptik, Germany). After inspection and development assessment, each sub-sample was returned to its incubator.

### Determination of ploidy

At hatching (approx. 96 hpf) samples of control and UV treated groups were collected and prepared for flow cytometric analyses. For this purpose, individual hatched larvae were gently rinsed in distilled water and placed at the bottom of a 1.5 ml Eppendorf tube. They were then dissociated by repeated manual pipetting in 1 ml of 0.05% Propidium Iodide (PI) solution, following established procedures [47]. After 30 min of PI staining in darkness at 4°C, 10% dimethyl sulfoxide (DMSO) was added and samples were stored at −80°C until use. Flow cytometry analyses were performed using a FACS Canto II (BD Biosciences, San Jose, CA, USA) flow cytometer and measuring the fluorescence of 5000 to 10000 nuclei/larva. The ploidy status of at least 20 hatched larvae (when available, or all surviving larvae in case of a lower number) from UV-treated groups and 10 control larvae was determined in each experiment.

### Microsatellite analysis

Verification of paternal inheritance was performed on presumptive androgenetic larvae coming from three different egg batches. For this purpose, 24 ml of eggs from three dams were UV-irradiated separately for 1 minute using the best performing UV dose (60 mJ.cm^−2^). After irradiation, the eggs were fertilized using the sperm of one of two sires (FAxM1; FBxM1; FCxM2) and putative androgenetic progenies incubated until hatching as previously described (see section 2.3). Individual hatched larvae were stored in absolute ethanol until genetic analyses. DNA was extracted from ethanol-preserved fin clips of the parent fish and from whole individual larvae using an E-Z 96 Tissue DNA Kit (Omega Bio-tek, Norcross, GA, USA) following the manufacturer’s protocol. Parental inheritance was assayed at 9 microsatellite loci: *Labrax*-17, *Labrax*-29, *Labrax*-3, *Labrax*-8 [48], *Dla*-22 [49], *Dla*-3 [50], *Dla*-16, *Dla*-105, *Dla*-119 [51] found on 9 different linkage groups (LG), these being LG23, LG18, LG13, LG16, LG6, LG19, LG1, LG8, LG14 respectively [52]. Forward primers were labeled with fluorescent dyes (Applied Biosystems). PCR reactions were carried out in 2.5 µl total volume containing 50–100 ng DNA, 0.1–1.0 µM of each primer set, 2x Qiagen Multiplex PCR (3 mM MgCl_2_, 6 U HotStarTaq DNA polymerase) and RNA-free water. DNA amplifications and PCR were performed on a GeneAmp PCR System 9700 (Applied Biosystems). The cycling program began with a polymerase activation step at 95°C for 15 min followed by 37 cycles of 94°C for 30 s, 59°C for 90 s and 72°C for 90 s, with a final extension at 72°C for 10 min. The PCR products were electrophoresed in a 3130x Genetic Analyzer (Applied Biosystems) and alleles scored using a GeneMapper Software v3.7 (Applied Biosystems).

### Spectrophotometry analyses

Egg pools from three females were UV-irradiated (see section 2.3) using four different incident dose rates (16.8, 30, 60 and 75 mJ.cm^−2^.min^−1^) and durations according to the following combinations: lower dose rates (16.8 and 30 mJ.cm^−2^.min^−1^) for 1, 2–12 min at 2 min intervals, and high dose rates (60 and 75 mJ.cm^−2^.min^−1^) for 0.5, 1, 2–12 min at 2 min intervals. Unirradiated eggs were used as controls. Immediately after treatment, samples of approximately 1500 eggs (1.5 ml egg volume) from treated and control groups were fixed in 96% ethanol and stored refrigerated until spectrophotometric analysis. Egg extracts from ethanol-stored samples were centrifuged at 2000 rpm for 4 min with a relative centrifugal force (RCF) of 1945 and 0.5 ml of the supernatant was diluted 1∶2 in 96% ethanol and scanned at wavelengths of 200 to 700 nm using a Hitachi U-2900 Double Beam Spectrophotometer (Hitachi High Technologies Corporation, Tokyo, Japan). Quartz cuvettes with 1 cm light path were used throughout the analyses. One control sample was analyzed using the same protocol but at lower pH (pH 3) obtained by addition of hydrochloric acid before wavelength scanning. UV absorbance raw data are available from the Dryad Digital Repository: http://doi.org/10.5061/dryad.k7s8s.

### Extraction and Ultra High Performance Phase Liquid Chromatography (UHPLC) analyses

Extraction was performed on 250 mg freeze-dried sea bass eggs in a 15 ml cuvette with 5 ml of methanol/water (50/50, v/v) (analytical grade, Sigma-Aldrich, St-Quentin Fallavier, France), assisted by sonication during 10 min. The sample was then centrifuged 5 min at 10,000 rpm and the final extract diluted in Acetonitrile (ACN) (1/1, v/v) (analytical grade, Sigma-Aldrich, St-Quentin Fallavier, France).

Ultra-high performance liquid chromatography (UHPLC) analyses were performed by a Dionex UltiMate 3000 RSLC system (Thermo Fisher Scientific, Waltham, USA) equipped for separation with a Kinetex HILIC (1.7 µm, 2.1×100 mm) (Phenomenex, Le Pecq, France) maintained at 40°C. The mobile phases consisted on (A) 10 mM Ammonium acetate (HPLC grade, Sigma-Aldrich, St-Quentin Fallavier, France) and (B) ACN (HPLC grade, Sigma-Aldrich, France) at constant flow-rate of 0.4 ml.min^−1^ (with gradient conditions described in File S2).

Metabolic fingerprints were measured using a Dionex UltiMate 3000 RSLC system coupled to an AB SCIEX TripleTOF 560 quadrupole-time-of-flight mass spectrometer (AB SCIEX, Concord, ON, Canada). Mass-spectrometric analysis was performed using an electrospray ion source (ESI) in both positive and negative ion mode. In the positive ESI mode, parameters were: capillary voltage of 4500 V, nebulizing gas pressure of 60 psi, drying gas pressure of 60 psi, temperature of 550°C and declustering potential of 80 V. The capillary voltage in negative ESI was −4000 V and the other source settings were the same as for positive ESI. Information Dependent Acquisition (IDA) method was employed to collect MS and MS/MS accurate mass. TOF MS and TOF MS/MS were scanned with the mass range of *m/z* 80–1200.

Instrument control and data acquisition were carried out with the Analyst 1.5.1 TF software (AB Sciex, Concord, ON, Canada) and the analysis was performed using Peak View 2.0 (AB Sciex, Concord, ON, Canada) also equipped with the MasterView Formula Finder and directly linked to ChemSpider database.

### Statistical analyses

At all stages, survival was estimated as a percentage of developing eggs over the total number of eggs. Survival rates were calculated relative to controls after adjustment of the latter to 100%. Survival rates were arcsin square roots transformed for comparison between groups by two-way ANOVA using Statistica (Version 7.1). Data are presented as means ± standard deviations (STD). Presence of null alleles in the PCR products was analyzed using Microchecker software version 2.2.3 [53].

## Results

### Embryonic and larval survival

**Figure 1:**
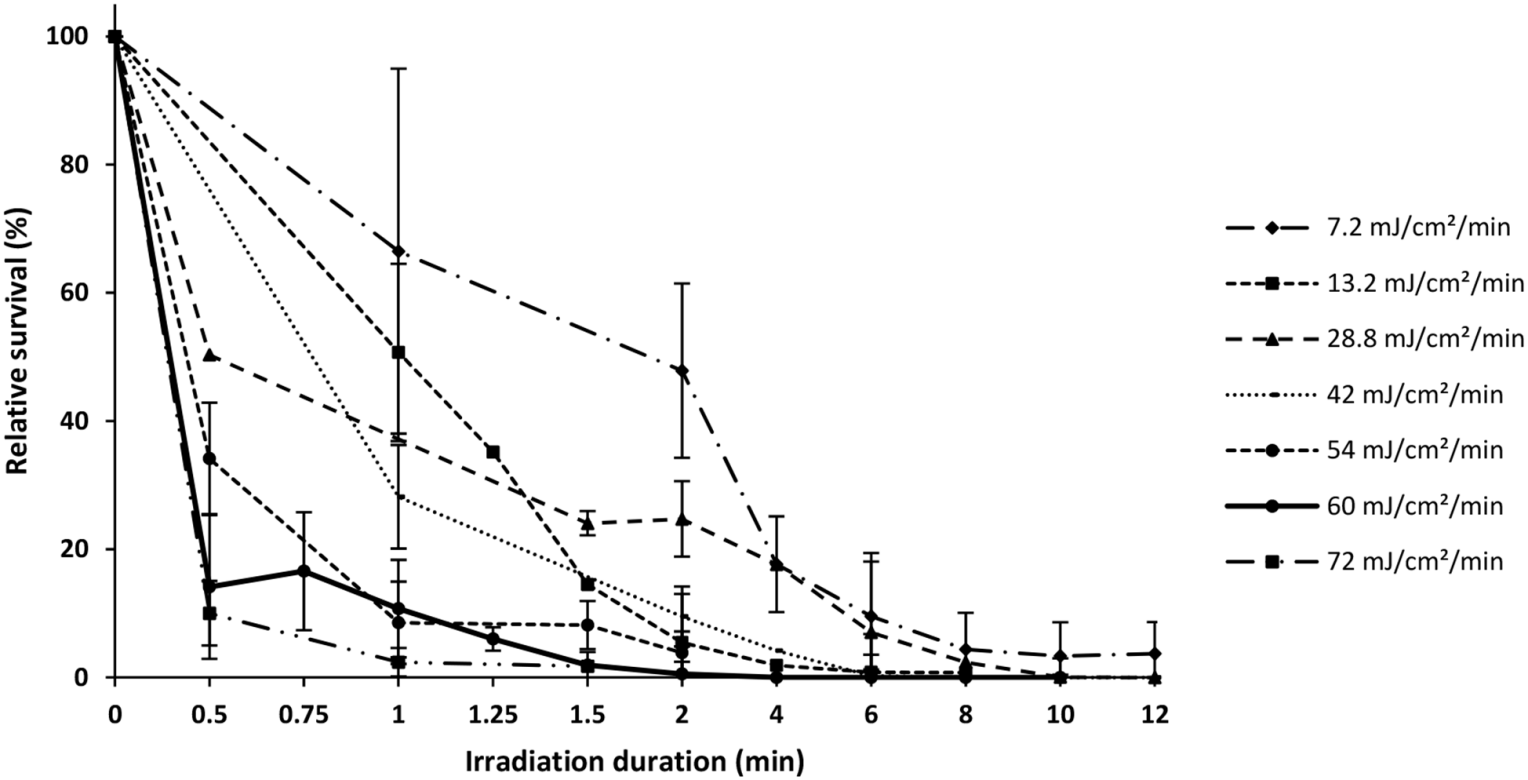
Percent survival relative to controls of hatched larvae issued from the different UV-irradiation treatments (7.2–72 mJ.cm^−2^.min^−1^) lasting 0.5–12 min. Error bars represent standard deviations of means (STD).

Overall, fertilization rates in the controls and the different treatments ranged from 20–80%, decreasing significantly at higher UV dose rates and longer durations (F_4,202_ = 22.344; F_7,202_ = 3.13; p<0.01), but with no interaction between the two factors (p = 0.11). Only survival rates relative to controls at 50 hpf are presented as no differences between data from 50 hpf and 74 hpf was observed (p = 0.47). Survival rates showed dose rate and duration effects (F_4,40_ = 254.67; F_7,40_ = 2695.7; p<0.001) and an interaction between these two factors (F_28,40_ = 18.48; p<0.001). Overall, larval survival fell sharply with increasing UV intensities and durations, in particular at the highest intensities (42, 60 and 72 mJ.cm^−2^.min^−1^) where survival dropped to 20% relative to controls after 1 min irradiation only (Fig. 1). At the lowest intensities (7.2 to 29 mJ.cm^−2^.min^−1^) survival rate decreased to less than 10% when eggs were irradiated up to 6 min, before reaching 0% between 10 and 12 min.

**Figure 2:**
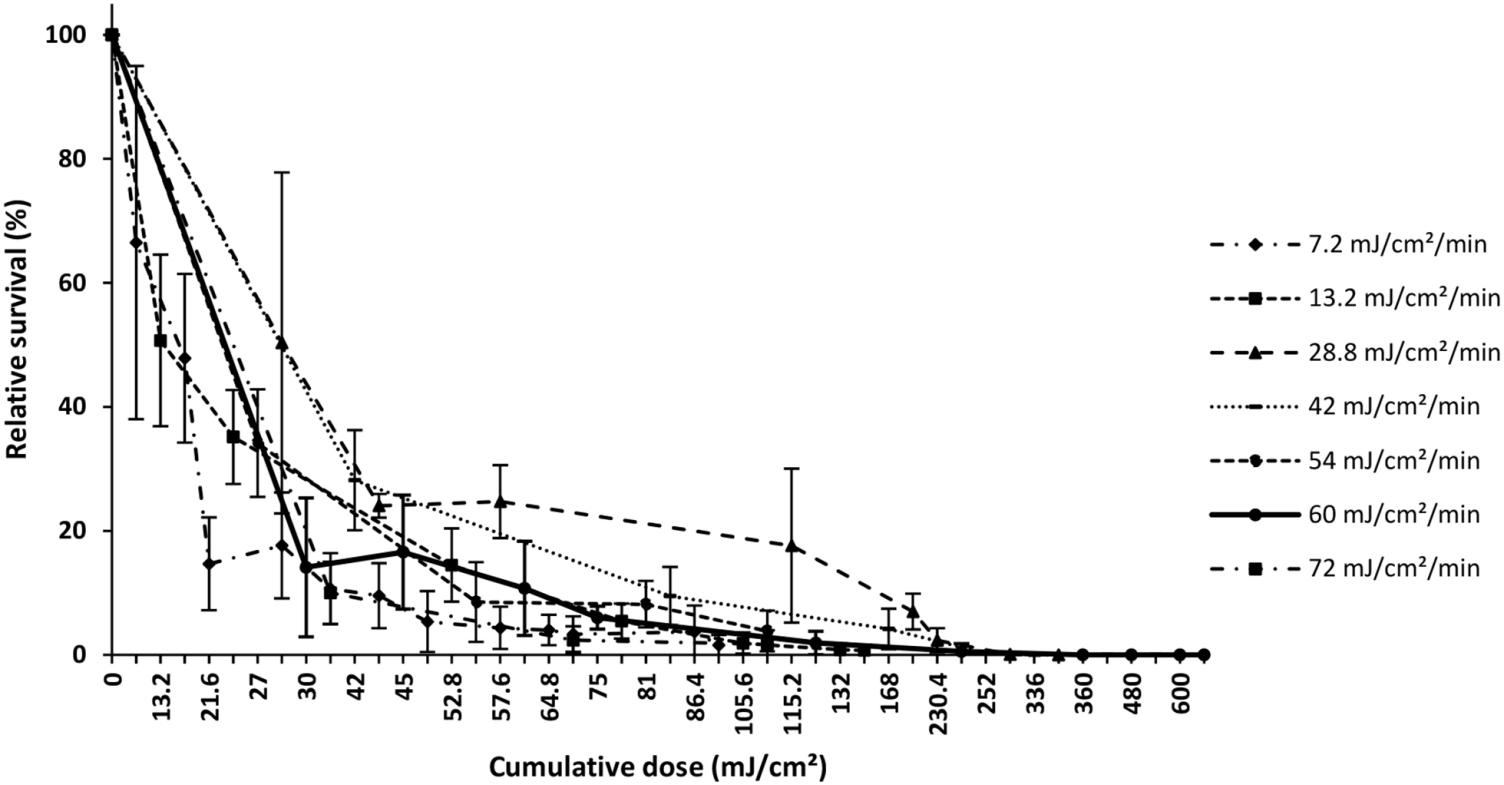
Percent survival relative to controls of treated groups exposed to different cumulative UV-doses in the range 7.2–720 mJ.cm^−2^. Error bars represent standard deviations of means (STD).

Cumulative UV doses (Fig. 2) showed a decrease in relative survival rates to 20% or less around 60 mJ.cm^−2^ before reaching 0% beyond 240 mJ.cm^−2^.

### Ploidy analysis

**Figure 3:**
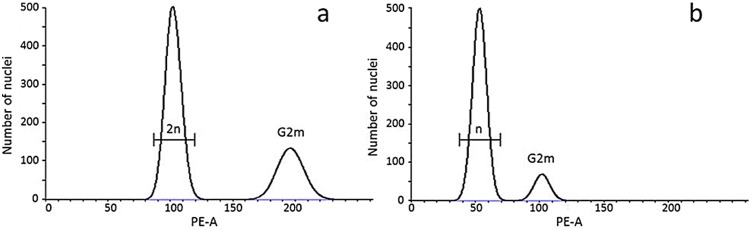
Representative examples of flow-cytometry histograms obtained from nuclear suspensions (5–10,000 counts) of Propidium Iodide (PI) stained larval samples. a) Control diploid (2n) larva (CV: 5%); b) haploid (n) larva produced with a UV-dose of 60 mJ.cm^−2^ (CV: 10%). DNA values on the X-axis are reported in arbitrary units expressed as fluorescent channel numbers (PE-A). G2 represent mitotic peaks.

Flow-cytometry analyses showed that only one UV treatment (60 mJ.cm^−2^.min^−1^ for 1 min) resulted in a small percentage (14%) of haploid larvae at hatching (Fig. 3). Overall, this corresponded to 3 haploids out of 21 hatched larvae and a yield of 1.4%. The analyses revealed that all other UV-treatments were ineffective at inactivating the maternal genome, yielding diploid larvae only.

**Figure 4:**
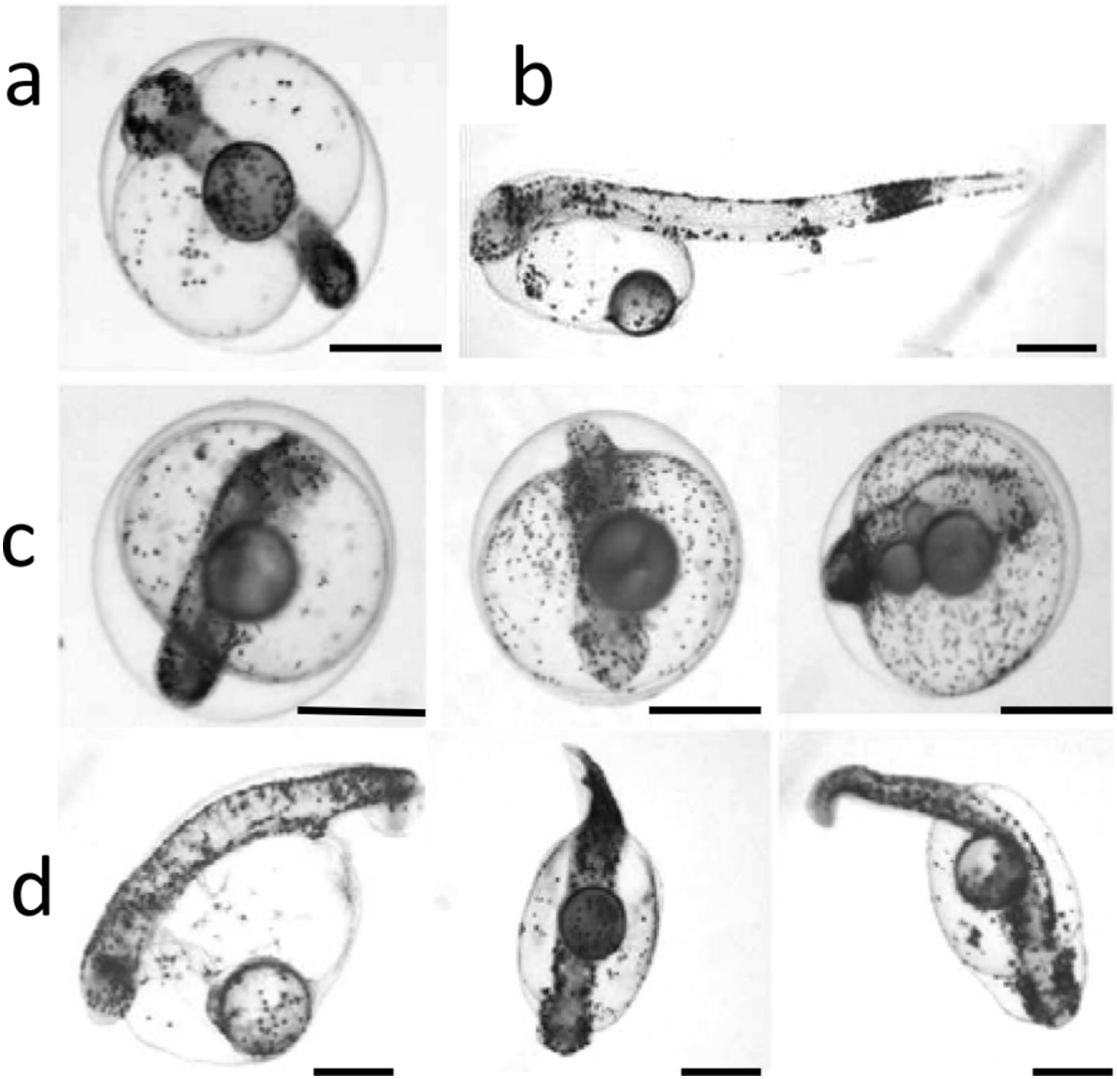
Morphology of control and UV-treated embryos and larvae. a) Control embryo at 74 h pf; b) control larva at hatching; c) UV treated embryos at 74 hpf showing microcephaly, short and large body; d) UV-treated larvae at hatching showing microphtalmy, short body and curved tail. Scale bars represent 500 µm.

UV irradiation at nearly all doses generated a wide range of deformities, including variable proportions of abnormal embryos and larvae which were morphologically similar to haploids. Typical ‘haploid syndrome’ malformations included short, twisted or large bodies, curved tail, microphtalmy and microcephaly as illustrated in Fig. 4.

### Microsatellite analyses

The genetic analyses of the three progeny groups exhibited different types of microsatellite inheritance (Table 1). The progeny group A1 (FAxM1) showed 43 individuals inheriting both paternal and maternal alleles for the nine microsatellite markers analyzed and a single larva displaying exclusively the paternal allele at one locus only. Progeny group B1 (FBxM1) contained 44 larvae with both paternal and maternal inheritance, two larvae showing maternal contribution at one locus and one larva with only paternal alleles for each marker. The last of these was concluded to be an androgenetic haploid. Female C (FC) showed a null (non-amplifying) allele which was detected after analyzing the segregation profile of *Labrax*-29 in its progeny, under the assumption of Hardy-Weinberg equilibrium in the transmission of alleles. Progeny group C2 (FCxM2) showed 76 larvae having inherited paternal and maternal alleles at all markers, one individual showing only paternal inheritance at one marker (*Dla*-22), one individual showing only paternal inheritance for at least five markers and one individual showed an unexpected genotype for *Labrax*-29, displaying both paternal alleles at this locus.

**Table 1:** Microsatellite marker loci transmission in three putative androgenetic progenies (A1, B1 and C2) produced with a UV-dose of 60 mJ.cm^−2^.

Fish	N	Marker loci								
		*Dla-22*	*Labrax-17*	*Labrax-29*	*Labrax-3*	*Dla-3*	*Dla-16*	*Dla-105*	*Dla-119*	*Labrax-8*
Female A		252/254	116/132	137/155	172/174	210/218	243/253	151/173	235/235	198/198
Male 1		230/254	116/138	133/159	136/190	216/218	237/245	157/171	225/257	198/232
Progeny A1	1	230/252	132/138	137/159	174/190	218/218	237/243	151/157	**257**	198/198
	43	bi-parental contribution at all loci								
Female B		248/252	116/134	131/155	130/176	216/228	239/241	137/145	227/257	212/212
Male 1		230/254	116/138	133/159	136/190	216/218	237/245	157/171	225/257	198/232
Progeny B1	1	**230**	116	**133**	**136**	**218**	**237**	**171**	**225**	**198**
	1	**230**	**138**	**159**	**190**	216	237/241	**157**	257	**232**
	1	**230**	116	**159**	**136**	**218**	237/241	**157**	257	**232**
	44	bi-parental contribution at all loci								
Female C		252/258	118/120	137/0	136/170	216/220	247/255	155/167	221/225	212/212
Male 2		236/254	118/138	133/159	116/136	220/226	231/265	157/157	227/235	222/232
Progeny C2	1	252/254	**138**	133/0	136	**226**	247/265	**157**	**235**	**232**
	1	254/258	118/120	**133/159**	136	216/220	231/255	155/157	225/227	212/222
	1	**236**	118/138	159/0	116/136	216/220	255/265	157/167	221/235	212/232
	76	bi-parental contribution at all loci								

Genotypes of progenies showing only discriminant paternal alleles are presented in bold characters. For putative androgenetic progenies, homozygous or haploid alleles are only written once since genotyping cannot distinguish between the presence of one or two copies of the same allele. N represents the number of analyzed individuals in each progeny.

### Characterization of potential UV screening compounds

**Figure 5:**
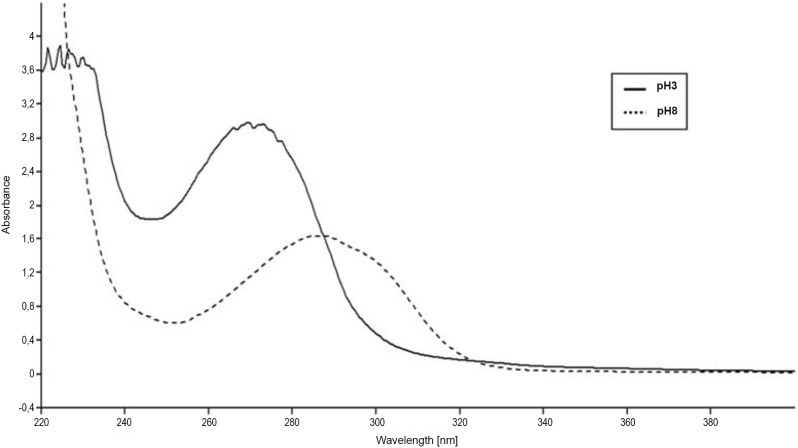
Absorbance spectrum of non-irradiated (control) egg extracts at pH8 (dotted line) and pH3 (solid line). Wavelengths cover almost the entire UV spectrum (UVC: 200–280 nm, UVB: 280–315 nm, and UVA: 315–400 nm). Absorption is shown as arbitrary units.

Spectrophotometry results showed the same wavelength scan curve for egg extracts from unirradiated controls and all combinations of UV dose rates and durations. The absorbance profiles covered the entire UV spectrum, with peaks of absorption typically around 285 nm and 269.5 nm at pH = 8 and pH = 3, respectively. A representative absorbance profile of egg extracts from the control group is shown in Fig. 5.

Gadusol, formula 5,6-Trihydroxy-5-(hydroxymethyl)-2-methoxy-2-cyclohexen-1-one (File S3), was found in the sample isolated from sea bass eggs at retention time (RT) 4 min, 203.0563 Da, using mobile phase B and the negative ionization mode (File S4).

## Discussion

The present work indicated that UV-irradiation was largely ineffective at inactivating the maternal genome in European sea bass eggs. Despite the wide range of UV doses employed (7.2–720 mJ.cm^−2^), only a small percentage of haploid androgenetics was produced at one of the doses tested. The different treatments covered UV dosages proven to be successful in freshwater species. For example, a UV dose of 45 mJ.cm^−2^ was effective at inactivating maternal DNA in *O. niloticus* [22], [23]. In the zebrafish, *D. rerio*, the optimal UV dose to induce haploid androgenesis was 144 mJ.cm^−2^ [24], while in the common carp, *C. carpio*, UV-doses of 250 mJ.cm^−2^ [27] and 175 mJ.cm^−2^ [36] have been successfully employed to inactivate maternal DNA. In the European sea bass, a marine species, the only dose that led to small proportions of verified haploids was 60 mJ.cm^−2^ and the androgenetic status of progenies produced at this dosage was tested using flow cytometry and DNA markers. Differential susceptibility to UV-irradiation among fish species may be attributed to dissimilarities in the thickness, composition and optic qualities of the egg chorion. Other factors may include differences in egg size and shape, and the relative position of the female pronucleus, making it difficult to compare egg irradiation treatments across species [23]. Different methods have been employed to achieve uniform UV treatment including manual or mechanical stirring of eggs kept in ovarian or synthetic fluids during irradiation from single or multiple UV-sources. In this work, we employed double UV sources (below and above) along with mechanical rotation of the eggs in order to maximize the efficiency of the treatment. The suitability of this purpose-built UV device was tested using the eggs of Nile tilapia as a positive control and haploid larvae were produced (Files S5 and S6) according to previously reported results in this species [22], [23].

In attempts at androgenesis in other species, the use of γ-rays and X-rays led to the typical ‘Hertwig effect’ and such paradoxical recovery in survival rates at high irradiation doses employing UV-rays was described in the Tiger barb, *Puntius tetrazona* [54] and the common carp [23], [27]. In other species, survival rates decreased with increasing UV intensities [30], [34], [55], [56]. The genotyping results highlighted some degree of variability in the response of eggs to UV treatment and corresponding androgenetic yield, possibly ascribed to egg quality factors. Myers et al. [23] showed evidence of female differential susceptibility to UV treatment affecting the yield of androgenetic haploids, but the mechanisms involved were not identified.

In the present study, sea bass embryos and larvae from UV-irradiated eggs possibly suffered partial denaturation of maternal genome and showed impaired development with a range of deformities similar to haploid syndrome. Similar results were observed in other species [24], [30], [33], [34] where larvae showed severe abnormalities like dwarfing, microcephaly, micropthalmy in most UV-treatments, even at low UV doses. In this experiment, flow cytometry and genotyping were employed to assess the ploidy status and genetically characterize hatched larvae derived from irradiated eggs. At the most efficient dose (60 mJ.cm^−2^), genotyping showed a vast majority of larvae with both paternal and maternal inheritance (biparental diploids), and only one larva (progeny B) with pure paternal inheritance (androgenetic haploid) and various levels of maternal inheritance in the remaining ones. A few larvae showed only paternal inheritance for one or a few markers (progeny A1 and C2): these individuals may have been aneuploids (near diploids) lacking one or a few chromosomes or fragments. In progeny B1, conversely, two larvae showed biparental inheritance for one or a few markers: these individuals may have been aneuploids, near haploids. These kinds of individuals could not be distinguished from real haploids or diploids using flow-cytometry (which did not show any sign of aneuploidy). Chromosome analyses are generally described as superior to flow cytometric methods because chromosome fragments and single chromosomal aneuploidy can be unambiguously detected [33]. However, in most studies dealing with haploid androgenesis, aneuploidy was observed for low UV doses which were inefficient at inactivating the maternal nuclear DNA. Also, the frequency of chromosome fragments and aneuploids decreased with increasing UV doses and only haploids were produced once the efficient UV dose was reached or exceeded [30], [33], [34].

The individual possessing both paternal alleles at one marker (*Labrax-29*) could have resulted from fertilization with an unreduced spermatozoon involving a single chromosome on which the heterozygous locus was located (LG28), the remaining microsatellite marker loci belonging to different linkage groups [52]. Although this remains a rare phenomenon, a small percentage (up to 1.6%) of aneuploid sperm has been previously reported for rainbow trout [57].

Several hypotheses can be put forward to explain the low success in inducing haploid androgenesis in the European sea bass. The first is the expression of recessive paternal alleles inducing high mortality at the homozygous state. Recessive mutations have been demonstrated to be one of the explanations for low survival rates of androgenetic and gynogenetic progenies in a number of species. Ungar et al. [24] showed that UV-irradiation of the maternal genome in zebrafish eggs uncovered recessive paternal mutations at the *gol* and *oep* loci at high frequency. Bertotto et al. [40] found one marker allele transmitted with a significantly lower frequency than the other in a mitotic gynogenetic progeny of *D. labrax*, suggesting a linkage to a deleterious gene. Another possibility for the low haploid yield in our work is impaired development and mortality due to the presence of maternal chromosome fragments. Chromosome fragments, probably of maternal origin, are considered to be a consequence of suboptimal UV treatment conditions and are more frequently reported in androgenetic than in gynogenetic progenies [33]. For example, interference of maternal DNA residues (participation in mitotic divisions) could be one reason for the poor viability of androgenetic muskellunge (*Esox masquinongy*) [55] and loach (*Misgurnus anguillicaudatus*) [34]. The presence of DNA fragments has been suggested as possible cause of the residual heterozygosity observed in diploid androgenetics of common carp although the maternal origin of these fragments could not be proved beyond doubt [36].

Another hypothesis for the low yield of haploid androgenetics based on the findings of the present study is the possible presence of some defense mechanisms against UV-irradiation in sea bass eggs. Screening compounds are known to provide a first line of defense in fish eggs [58],[59] while active DNA repair processes may be used by eggs to deal with damage caused by UV [60]. Photoreactivation and dark repair pathways are known processes for fixing or replacing UV-damaged DNA. In order to prevent activation of DNA-repairing mechanisms under the influence of visible light in the laboratory, the egg irradiation procedures are commonly completed under total darkness. In our case, the application of dark conditions during egg irradiation and early incubation should have prevented the possibility of light-dependent mechanisms being activated. Nevertheless, as these mechanisms can never be 100% efficient, many organisms naturally exposed to UV radiation for parts of their life-cycle can passively screen UV radiation to prevent its potential damage in the first place [58]. In fish, UV-screening compounds such as gadusol and related mycosporine-like amino acids (MAAs) are found in the eggs of Atlantic cod and other marine teleost [42], [43], [61]. In particular, gadusol shows strong absorption towards the UV-B and UV-C spectrum with pH-dependent distinctive maxima: λ_max_ (H_2_O, pH<2)/nm 269 (ε/dm^3^ mol^−1 ^cm^−1^ 12400) and 296 (21800) at pH>7 [43], [62]. Differential absorption pH-dependent was also observed in our experiment and HPLC characterization confirmed the presence of gadusol in sea bass eggs. European sea bass eggs are small, transparent and UV screening compounds like MMAs are present as observed in other marine fish producing comparable eggs (e.g. Atlantic cod). Based on our attempts at maternal genome inactivation and preliminary assessment of UV absorbance by the extracts of sea bass eggs, this last hypothesis seems plausible. Further work on the comparison of UV absorbance and chemical characterization of putative UV screening compounds like gadusol in the eggs of this and other marine species with those of freshwater species where androgenesis has been successfully reported would allow testing of this hypothesis.

If the eggs of such marine species are protected against UV, then ionizing radiation, although more difficult to work with than UV, might be more effective in successfully inducing haploid (and diploid) androgenesis in the European sea bass and other marine species. A novel method aimed at inducing androgenesis in the eggs of freshwater fish without the use of irradiation was reported by Morishima et al. [63]. These authors succeeded in producing relative high percentages of haploid androgenetic embryos among the survivors of newly fertilized cold-shocked eggs of loach (*M. anguillicaudatus*). The treatment induced the extrusion of the egg pronucleus together with the second polar body, leaving only the sperm pronucleus in the egg. Further work on this ‘cold-shock technique’ [64] focused on the production of androgenetic diploid loach embryos, and yielded approximately 10% diploid androgenetic larvae as well as proportions of haploid, triploid, tetraploid, pentaploid, aneuploid and mosaic larvae. Despite these constraints, the method may represent an alternative to the UV-irradiation of eggs and may be worth exploring for the induction of androgenesis in European sea bass. Another alternative method for the production of androgenetic progenies in the European sea bass could be interspecific androgenesis. The use of egg donors has been attempted in several freshwater species and resulted in varying success. The first successful attempt was the production of androgenetic goldfish (*Carassius auratus auratus* L.) using common carp (*Cyprinus carpio* L.) eggs [65]. Brown & Thorgaard [66] reported androgenetic development of rainbow trout (*O. mykiss*) with Yellowstone cutthroat trout (*Oncorhynchus clarki bouvieri* R.) eggs and more recently, androgenetic common tench (*Tinca tinca* L.) developed from common carp and common bream (*Abramis brama* L.) eggs [67]. Other experiments of interspecific androgenesis between salmonids [68] and sturgeons [69] led to inviable androgenetic progenies though viable hybrids could be produced. These results suggest that interspecific androgenesis is possible only between closely related species showing similar karyotypical characteristics [68], [69]. To avoid nucleocytoplasmic incompatibility, interspecific androgenesis can be achieved using as egg donor a hybrid of the species whose sperm is used for fertilization. Accordingly, viable androgenetic carps were obtained from eggs derived from the goldfish x carp hybrid females [70], brook charr (*Salvelinus fontinalis* M.) x Arctic charr (*Salvelinus alpinus* L.) hybrid eggs were used to induce androgenesis in brook charr and resulted in small percentages of diploid androgenetic larvae [71]. Though the nucleocytoplasmic compatibility of European sea bass sperm with eggs from another species in which androgenesis was successful is not granted, this approach could be explored as possible alternative for the induction of androgenesis in sea bass.

## Supporting Information

File S1 **Positive control for UV-irradiation device.**
(DOCX)

File S2 **HPLC elution gradient used for the separation of metabolic fingerprints.**
(DOCX)

File S3 **Chemical structure of gadusol. λmax = 268 nm at pH 2.5, λmax = 294 nm at pH 7.**
(TIF)

File S4 **Spectra obtained from HPLC analyses for the identification of gadusol.** a) Extracted ion chromatogram for gadusol, m/z 203.0561 retention time 4 min. b) TOF MS spectrum from 4.154 to 4.176 min. c) TOF MS/MS spectrum from 4.068 min.
(TIF)

File S5 **Representative examples of flow-cytometry histograms obtained from nuclear suspensions (5–10000 counts) of Propidium Iodide (PI) stained** ***O. niloticus*** **larvae**. a) Control diploid (2n) larva (CV: 6.5%); b) haploid (n) larva produced with a UV-dose of 42 mJ.cm^−2^ (CV: 7%). DNA values on the X-axis are reported in arbitrary units expressed as fluorescent channel numbers (PE-A). G2 represents mitotic peaks.
(TIF)

File S6 **Androgenesis in Nile tilapia,** ***O. niloticus*** **.**
(DOCX)
