# Supplementary material for: Genetic Inactivation of European Sea Bass (Dicentrarchus labrax L.) Eggs Using UV-Irradiation: Observations and Perspectives
Source: PLoS One. 2014 Oct 20;9(10):e109572. doi: 10.1371/journal.pone.0109572 (PMC4203730; doi:10.1371/journal.pone.0109572)
Supplement: File S1 — Positive control for UV-irradiation device. (DOCX) [file pone.0109572.s001.docx]

The efficiency of the UV-device (section 2.2) was tested on Nile tilapia, a species for which the successful induction of androgenesis has been reported [[1](#_ENREF_1),[2](#_ENREF_2)]. For this purpose, domesticated broodstock *O. niloticus* of Bouaké strain aged 3 years and weighting 150 to 250 g were obtained from Cirad (Montpellier, France) and transported to Ifremer Palavas-les-Flots where they were maintained and handled using standard hatchery strip-spawning techniques. A single ready to spawn female was stripped and the egg batch (around 1000 eggs) equally divided into individual Petri dishes, diluted in 10 ml freshwater and UV irradiated at 10.5 mJ.cm^-2^.min^-1^ for 0, 1, 2, 4 or 6 min. UV treated and control (unirradiated) eggs were fertilized, after removal of water, by adding 200 µl sperm diluted 1:10 in Modified Fish Ringer’s solution (NaCl, KCl, NaHCO_3_ and CaCl_2_) [[3](#_ENREF_3)] and activated by addition of freshwater 1 min later. Treated and control eggs were reared in individual flow-through 2 L incubators at 28°C until hatching (96 hpf). All hatched larvae from UV and control treatments were rapidly separated from the yolk sac under a dissecting microscope and treated for flow cytometry as described previously (section 2.5)

**References**

1. Karayucel S, Karayucel I, Penman D, McAndrew B (2002) Production of androgenetic Nile tilapia, *Oreochromis niloticus* L.: optimization of heat shock duration and application time to induce diploidy. Israeli Journal of Aquaculture-Bamidgeh 54: 145-156.

2. Myers JM, Penman DJ, Basavaraju Y, Powell SF, Baoprasertkul P, et al. (1995) Induction of diploid androgenetic and mitotic gynogenetic Nile tilapia (*Oreochromis niloticus* L.). Theoretical and Applied Genetics 90: 205-210.

3. Sarder MRI (1998) Genetic analysis of specific and non-specific immune response in *Oreochromis niloticus* L. Ph.D. Thesis. University of Stirling.
