# Supplementary material for: Genetic Inactivation of European Sea Bass (Dicentrarchus labrax L.) Eggs Using UV-Irradiation: Observations and Perspectives
Source: PLoS One. 2014 Oct 20;9(10):e109572. doi: 10.1371/journal.pone.0109572 (PMC4203730; doi:10.1371/journal.pone.0109572)
Supplement: File S2 — HPLC elution gradient used for the separation of metabolic fingerprints. (DOCX) [file pone.0109572.s002.docx]

| Retention time (min) | Flow rate (ml.min^-1^) | % A | % B |
| --- | --- | --- | --- |
| Initial | 0.4 | 5 | 95 |
| 1 | 0.4 | 5 | 95 |
| 8 | 0.4 | 45 | 55 |
| 10 | 0.4 | 45 | 55 |
| 10.1 | 0.4 | 5 | 95 |
| 15 | 0.4 | 5 | 95 |
