# Supplementary figures and images for: Genetic Inactivation of European Sea Bass (Dicentrarchus labrax L.) Eggs Using UV-Irradiation: Observations and Perspectives
Source: PLoS One. 2014 Oct 20;9(10):e109572. doi: 10.1371/journal.pone.0109572 (PMC4203730; doi:10.1371/journal.pone.0109572)

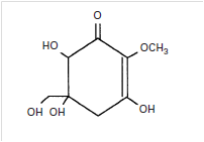

Supplement: File S3 — Chemical structure of gadusol. λmax = 268 nm at pH 2.5, λmax = 294 nm at pH 7. (TIF) [file pone.0109572.s003.tif]

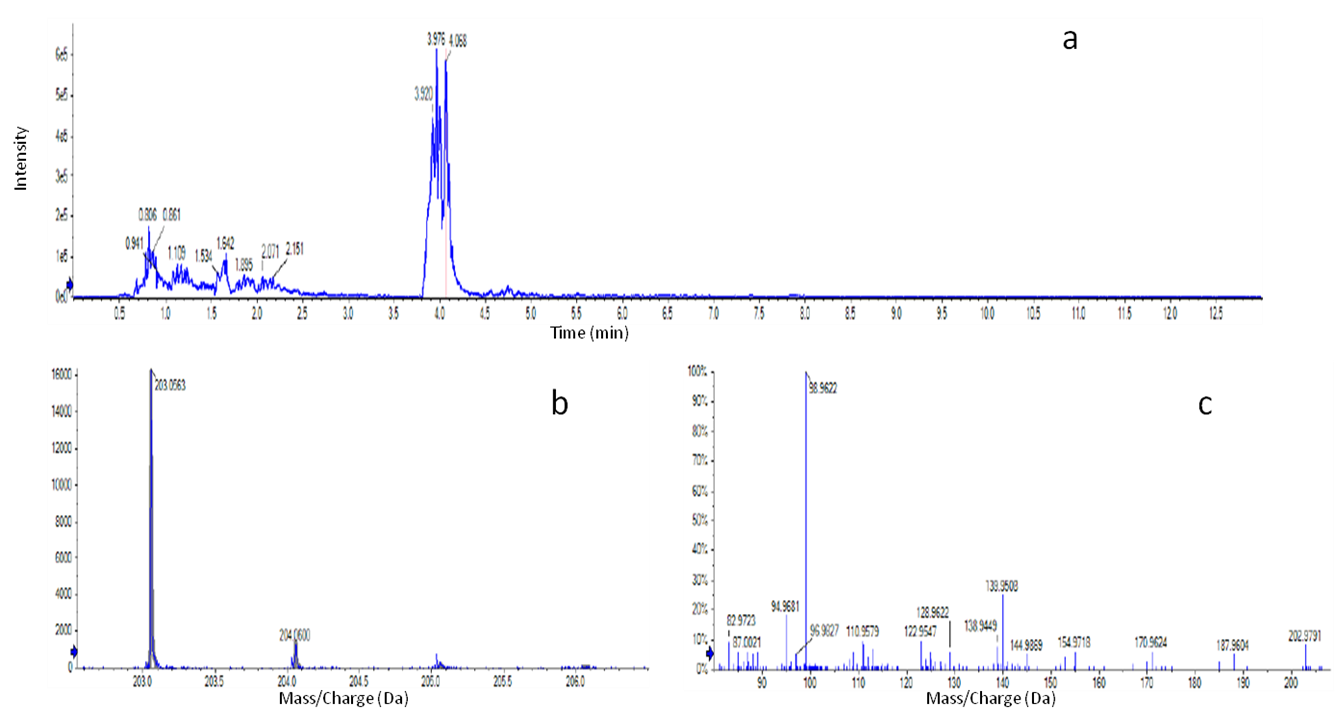

Supplement: File S4 — Spectra obtained from HPLC analyses for the identification of gadusol. a) Extracted ion chromatogram for gadusol, m/z 203.0561 retention time 4 min. b) TOF MS spectrum from 4.154 to 4.176 min. c) TOF MS/MS spectrum from 4.068 min. (TIF) [file pone.0109572.s004.tif]

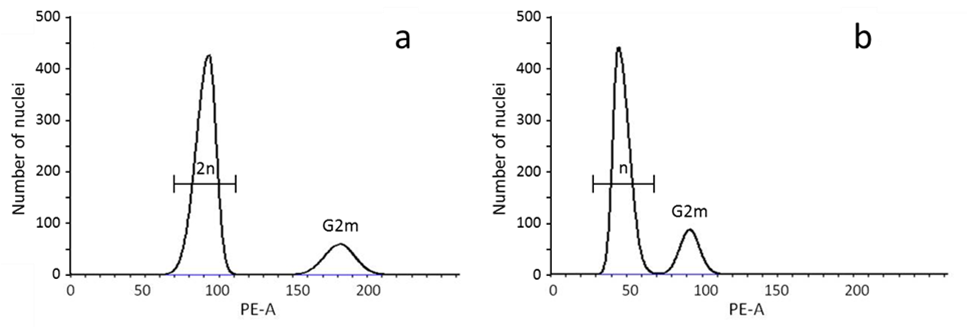

Supplement: File S5 — Representative examples of flow-cytometry histograms obtained from nuclear suspensions (5–10000 counts) of Propidium Iodide (PI) stained O. niloticus larvae. a) Control diploid (2n) larva (CV: 6.5%); b) haploid (n) larva produced with a UV-dose of 42 mJ.cm−2 (CV: 7%). DNA values on the X-axis are reported in arbitrary units expressed as fluorescent channel numbers (PE-A). G2 represents mitotic peaks. (TIF) [file pone.0109572.s005.tif]
