# Supplementary material for: Genetic Inactivation of European Sea Bass (Dicentrarchus labrax L.) Eggs Using UV-Irradiation: Observations and Perspectives
Source: PLoS One. 2014 Oct 20;9(10):e109572. doi: 10.1371/journal.pone.0109572 (PMC4203730; doi:10.1371/journal.pone.0109572)
Supplement: File S6 — Androgenesis in Nile tilapia, O. niloticus . (DOCX) [file pone.0109572.s006.docx]

Flow-cytometry analyses showed that 4 min irradiation with a UV dose rate of 10.5 mJ.cm^-2^.min^-1^ was an effective treatment to induce androgenetic haploids in the Nile tilapia (Fig. S4). Of the two surviving larvae at 96 hpf out of around 150 fertilized eggs, both were haploid. All control larvae assayed (n=10) were diploid, whereas 1 and 2 min irradiation resulted in four and one diploid larvae at hatching, respectively. After 6 min irradiation, no larval survival was observed.
